# Large scale newborn deafness genetic screening of 142,417 neonates in Wuhan, China

**DOI:** 10.1371/journal.pone.0195740

**Published:** 2018-04-10

**Authors:** Zongjie Hao, Denggang Fu, Yang Ming, Jinlong Yang, Qi Huang, Weilong Lin, Huan Zhang, Bin Zhang, Aifen Zhou, Xijiang Hu, Cong Yao, Yunping Dong, Huijun Z. Ring, Brian Z. Ring

**Affiliations:** 1 Institute of Genomic and Personalized Medicine, College of Life Science, Huazhong University of Science and Technology, Wuhan, Hubei, China; 2 Wuhan Women and Children Hospital, Wuhan, Hubei, China; 3 Health and Family Planning Commission of Wuhan Municipality, Wuhan, Hubei, China; German Cancer Research Center (DKFZ), GERMANY

## Abstract

Almost one third of the three million people in China suffering severe deafness are children, and 50% of these cases are believed to have genetic components to their etiology. Newborn hearing genetic screening can complement Universal Neonatal Hearing Screening for the diagnosis of congenital hearing loss as well as identifying children at risk for late-onset and progressive hearing impairment. The aim of this joint academic and Ministry of Health project was to prototype a cost effective newborn genetic screen in a community health setting on a city-wide level, and to ascertain the prevalence of variation at loci that have been associated with non-syndromic hearing loss. With the participation of 143 local hospitals in the city of Wuhan, China we screened 142,417 neonates born between May 2014 and Dec. 2015. The variants GJB2 c.235delC, SLC26A4 c.919-2A>G, and mitochondrial variants m.1555A>G and m.1494C>T were assayed using real time PCR. Newborns found to carry a variant were re-assayed by sequencing in duplicate. Within a subset of 707 newborns we assayed using real-time PCR and ARMS-PCR to compare cost, sensitivity and operating procedure. The most frequent hearing loss associated allele detected in this population was the 235delC variant in GJB2 gene. In total, 4289 (3.01%) newborns were found to carry at least one allele of either GJB2 c.235delC, SLC26A4 c.919-2A>G or two assayed MT-RNR1 variants. There was complete accordance between the real-time PCR and the ARMS PCR, though the real-time PCR had a much lower failure rate. Real-time PCR had a lower cost and operating time than ARMS PCR. Ongoing collaboration with the participating hospitals will determine the specificity and sensitivity of the association of the variants with hearing loss at birth and arising in early childhood, allowing an estimation of the benefits of newborn hearing genetic screening in a large-scale community setting.

## Introduction

Hearing loss is one of the most common human disorders, and genetic causes contribute to more than half of congenital hearing loss cases[1, 2]. Although 30,000 neonates with hearing loss are identified each year in China via universal neonatal hearing screening, the number of people with hearing defects who are not registered as such by the government is far greater[3]. In addition, it has been shown that the prevalence of permanent non-syndromic hearing loss (NSHL) increases about 50 percent during childhood, and doubles during adolescence. This is due to delayed detection of congenital hearing loss, late-onset of hearing loss, and aminoglycoside-induced hearing loss[4, 5]. Early detection of hearing loss in newborns is very important, hearing-impaired neonates show improved outcome when their hearing loss is recognized before 6 months after birth[6–10]. Newborn deafness genetic screening can be a complementary tool to traditional physical hearing tests[11]. More than 20 cities in China have carried out newborn deafness genetic screening projects, but few large studies have been performed and the frequency of common hearing loss mutations across the Chinese population has not been well estimated[1, 2].

Deafness is characterized by its etiological heterogeneity. It is estimated that about 50% of cases of childhood hearing loss are associated with genetic factors. Loci in more than 70 genes have been found to be associated with NSHL[1, 2]. Additionally, genetic variants with known associations with congenital hearing loss have been identified[12, 13].Variations in GJB2, SLC26A4 and MT-RNR1 are the most common variants associated with NSHL in China[1]. Mutations in GJB2, encoding gap junction beta 2 protein (connexin 26), are the most common variants linked to non-syndromic hearing impairment worldwide[14–16]. However, the variants and their prevalence vary significantly in different ethnic populations[1, 17]. GJB2 c.35delG and GJB2 c.167delT are found to be the most frequent mutations in Caucasian and Ashkenazi Jewish groups, while GJB2 c.235delC is the most frequently seen mutation in East Asian populations[1]. GJB2 biallelic variants have been found in approximately 25% of infants diagnosed with hearing loss[17, 18]. Infants who failed newborn hearing screens were 11.8 times more likely to have carry GJB2 variants than infants who passed the hearing screen [17, 19]. Variations in SLC26A4 are the second most common genetic cause of sensorineural hearing loss[20], and are responsible for Pendred syndrome, an autosomal recessive disorder marked by enlarged vestibular aqueducts and concomitant sensorineural hearing loss. Mutations in this gene are associated with 3% of newborn incidences of NSHL, but the frequency increases significantly in later years[21], and appears to be associated with enlargement of the vestibular aqueduct[22]. SLC26A4 encodes a transmembrane exchanger of negative ions, and is expressed in the inner ear. The most common mutation of SLC26A4 found in the Chinese population is c.919-2A>G, its carrier frequency can be as high as 12.5%[1, 23]. Aminoglycoside antibiotics have been associated with high rates of nephrotoxicity and ototoxicity in some people, especially among carriers of variants in the mitochondrial 12S gene[24]. The mitochondrial variant m.1555A>G, though infrequent in a general population of NSHL cases[21], is the most common allele associated with aminoglycoside-induced deafness and NSHL in several ethnic groups, and m.1494C>T was the second most prevalent mutant[1, 25].

Early-detection is of vital importance in addressing the needs of newborns with hearing loss. The costs of identifying newborns with hearing loss via universal screening are relatively low[26–28], and can be economically beneficial even in developing countries[29]. Additionally, identification of mutations in mitochondrial genes associated with aminoglycoside-induced deafness can prevent the inappropriate use of these drugs. Studies have shown that newborn hearing concurrent gene screening was an effective complement to standard hearing assessments for the improved care of infants[30] and can identify children whose hearing loss occurs later in childhood[31]. It remains unanswered what is the most efficient and reliable means of identifying hearing loss related genetic variants in a public health effort. Next-generation sequencing (NGS) can be an effective tool for identifying known and novel variants related with hearing loss[32], but the cost and complexity of analysis may limit its applications to community level health care delivery, especially in developing regions. Chip based assays may be more cost effective, but it is difficult to add novel genetic loci related to hearing loss, the number of which are continually growing[30, 32]. PCR is highly cost effective for a small number of variants and samples, readily adoptable in a variety of health care delivery sites, and assays specifically targeted towards variants associated with NSHL have been approved for use in clinical diagnoses[33]. However standard PCR can have difficulties in scaling to large numbers of samples, though several different variants of the technique have been used in the screening of NSHL associated variants[34].

In the present study we performed one of the largest scale genetic screenings in a joint academic and Ministry of Health project, assaying 142,417 neonates born between May 2014 and December 2015 in Wuhan, China. 98.7% of newborns at the contributing 143 local hospitals participated in this screening during the time of the project. Deafness-related variants in three genes (GJB2, SLC26A4 and MT-RNR1 (mitochondrially encoded 12S RNA)) were assayed using real time PCR. The aim was to develop a low-cost, fast, accurate and high-throughput platform for large scale genetic screening applicable to a community health care setting and to compare to other methodologies. This also allows a comparison of the frequency of the chosen variants in Wuhan to other regions and to better assess the variants best suited for this assay.

## Materials and methods

### Recruitment of the subjects

The population enrolled in our study consisted of 142,417 newborns in Wuhan, which is located in the central of China. From May 2014 to Dec. 2015, 142,417 newborns were recruited from 143 hospitals, including both maternity and general hospitals. The participation rate was 98.7%. The screening protocol was approved by the Health and Family Planning Commission of Wuhan Municipality and all the participating hospitals. Three to four heel blood spots (diameter≥12 mm, 30–40 μL) were collected within 72 hours after birth according to standard protocol with FTA cards. The protocol was as follows: the heel of the foot was cleaned with an alcohol wipe, punctured at the edge of the plantar surface using an automated lancet, the first drop of blood was discarded, and a single blood spot was collected per card, and the cards were air dried four hours. The sampling card also contained the participants’ information (sample’s unique ID, newborn’s or mother’s name, sex and ethnicity, birth date, hospital, and blood collection date). All newborns’ information were gathered into a newborn deafness genetic database, and unused blood spots were stored as a biobank. Samples with inadequate number or size of blood spots, samples that were stored improperly, or samples with incomplete information were disqualified from analysis. Written informed consent was obtained from all the neonates’ parents or guardians who participated in the project. The study protocol was reviewed and approved by the internal review board of Huazhong University (#[S189]).

### Selection of variants

A review of published meta-analyses was used to nominate candidate variants. Criteria for inclusion were repeated strong associations with risk of NSHL, potential actionability upon diagnosis, and prevalence in East Asian populations. Variants in GJB2, SLC26A4, MTRNR1 were among the most commonly related to non-syndromic hearing loss[35–38]. The c.235delC mutation of GJB2 gene, the c.919-2A>G mutation of SLC26A4 gene and the MTRNR1 m.1555A>G and m.1494 C>T were selected for further study.

### DNA isolation and real-time PCR analysis

Genetics variants with known associations with hearing loss were assessed. The variants GJB2 c.235delC, SLC26A4 c.919-2A>G, and mitochondrial variants in the MT-RNR1 12S gene m.1555A>G and m.1494C>T were assayed using three independent fluorescent PCR kits. All variants were detected using a real time fluorescent PCR method with a commercial kit (Yingsheng, Jinan, China). The detection kit is comprised of genomic DNA extraction reagents and the fluorescent PCR reagents, including positive and negative control, and amplification reaction mixtures. All reactions for each variant were performed in a single well. Amplification was performed with ViiA^™^ 7 system on 384-well plates (ThermoFisher Scientific, Singapore). The genotypes of each allele were determined via amplification curves and the Ct value. Newborns found to carry a variant were confirmed by sequencing in duplicate. Heteroplasmy and homoplasmy of the mitochondrial variants were identified by the presence or absence of unique amplification curves.

### The Taqman-MGB genotyping platform

The specific Taqman-MGB probe sets for (GJB2 c.235delC, SLC26A4 c.919-2A>G, MT-RNR1 m.1555A>G and m.1494C>T) with wild and mutant type were designed using Primer 3.0 and validated by Sanger sequencing. Each loci required a pair of primers common to both wild type (P1) and the mutant sequences (P2), as well as two different MGB (Minor Groove Binder) probes for each assay. The probe for the normal sequence was labeled with the FAM fluorophore, and the probe for the mutant sequence was labeled with the VIC fluorophore. A pair of regular PCR primers (F1, F2) for each mutation is also designed at the same time.

Each PCR reaction was carried out in a total volume of 5 μl. The appropriate concentration of a mixture of probes and primers (mixture: primer F1, F2 and Taqman-MGB probes: P1, P2) was added at a volume of 0.25μl, along with 2.5 μl 2X PCR Buffer (GeneCore, China), 1μl genomic DNA template and 1.25μl ddH2O (Sangon Biotech, China) (Table 1).

**Table 1:** Primers and Taqman-MGB probes for the four mutations.

Genes	Mutations	SNP	Primers or Probes names	Primers or Probes sequences(5'-3')
**SLC26A4**	c.919-2A>G	rs111033313	SLC26A4-Forward	AAAGTTCAGCATTATTTGGTTGACAA
			SLC26A4-Reverse	TTCCAGGTTGGCTCCATATGA
			SLC26A4-P1	FAM-CATCTTTTGTTTTATTTCAGACG-MGB
			SLC26A4-P2	VIC-TCTTTTGTTTTATTTCGGACGA-MGB
**GJB2**	c.235delC	rs80338943	GJB2-235-Forward	TGGCGTGGACACGAAGATC
			GJB2-235-Reverse	CTACTTCCCCATCTCCCACATC
			GJB2-235-P1	FAM-CTGCAGGGCCCATA-MGB
			GJB2-235-P2	VIC-CTGCAGGCCCATAG-MGB
**MTRNR1**	m. 1555A>G	rs267606617	mit-1555-Forward	TGCACTTTCCAGTACACTTACCATGT
			mit-1555-Reverse	GCCCGTCACCCTCCTCA
			mit-1555-P1	FAM-ACGACTTGTCTCCTCTA-MGB
			mit-1555-P2	VIC-ACGACTTGCCTCCT-MGB
**MTRNR1**	m.1494C>T	rs267606619	mit-1494-Forward	GCCCTGAAGCGCGTACAC
			mit-1494-Reverse	CCATGTTACGACTTGTCTCCTCTATATAA
			mit-1494-P1	FAM-CGCCCGTCACCCT-MGB
			mit-1494-P2	VIC-CCGTCACTCTCCT-MGB

PCR amplification was commenced with an initial denaturation step at 94 °C for 10 min, followed of the cycling conditions and annealing temperatures indicated in Table 1. The final extension step was at 72 °C for 5 min. 707 randomly chosen samples were also assayed using a Tetra-primer ARMS PCR kit (BioSino Bio, Beijing, China) in order to validate and compare identification of the four mutations. Gel electrophoresis was used to distinguish the ARMS PCR products. Heteroplasmy and homoplasmy of the mitochondrial variants could not be distinguished in this manner, and sequencing was performed to distinguish these products (data not shown). Anonymized genotype results are found in the supplementary S1 Table.

### Sequencing validation

Additionally, all variants cases detected by these two PCR assays were validated via Sanger sequencing using sequencing primers designed with Primer 3.0 (Table 2). Three pairs of primers were designed and the targeted sequence was amplified. All the PCR products were purified on QIAquick Gel Extraction Kit (Qiagen, Valencia, CA) and subjected to direct sequencing by BigDye Terminator Cycle Sequencing kit (version v.3.1) and ABI genetic analyzer 3730. If the sequencing was discrepant from real time PCR result, the genotypes result from the sequencing would be reported.

**Table 2:** Sequencing primers for four NSHL associated loci.

Genes	Mutations	Primer names	Primer sequence(5'-3')	Bases(bp)	Purification
**GJB2**	c.235delC	GJB2-246-F1	AGAGTTGGTGTTTGCTCAGG	20	PAGE
		GJB2-1011-R1	TTCAGTGACATTCAGCAGGA	20	PAGE
**SLC26A4**	c.919-2A>G	SLC26A4-253-F1	GATTTCACTGCTGGATTGCT	20	PAGE
		SLC26A4-756-R1	GCATATACGGGCTGCTTTTA	20	PAGE
**MTRNR1**	m.1555A>G/m.1494C>T	MTRNR1-192-F1	TAATCGATAAACCCCGATCA	20	PAGE
		MTRNR1-761-R1	TATCTATTGCGCCAGGTTTC	20	PAGE

#### Statistical analysis

The inter-city differences in frequency of overall and single site carriers were compared using the two-tailed Chi-square test. A P-value less than 0.05 was considered statistically significant.

## Results

### The workflow of newborns deafness gene screening

A total of 142,417 neonates were enrolled into the newborn deafness genetic screening study, and a high throughput genetic screening standard procedure was needed to assay their genotypes. Via discussion with the Health and Family Planning Commission and a collaborating otolaryngologist, a workflow was proposed and tested on a pilot project of 500 participants. As the initial results were favorable, the project was expanded to the total size reported in this study.

**Fig 1:**
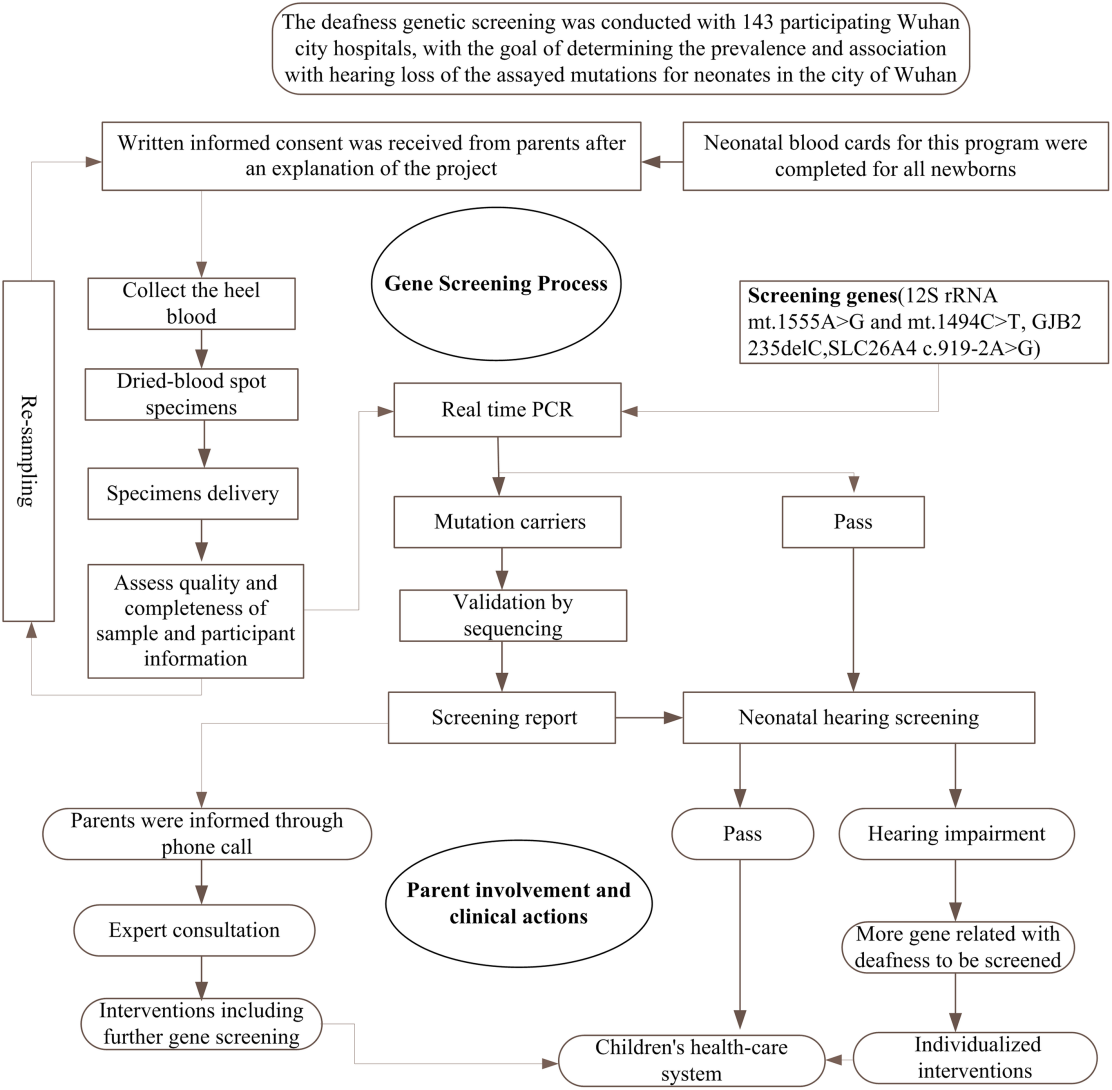
Flow chart of neonates’ deafness gene screening.

The protocol was comprised of four stages, including blood specimen collection, genetic screening, result interpretation, and follow-up intervention (Fig 1). All participants provided written informed consent before heel blood samples were collected. Three dried blood spots were collected and delivered to a single genetic screening center. Qualified samples were put into the genetic testing flow. Doctors and genetic counselors were responsible for interpreting the screened results and for suggesting possible intervention measures for mutation carriers. Those who carried alleles associated with increased risk of NSHL were registered with their local women and children health-care system for follow-up studies.

### Results of the genetic screening

Genetic screening data of four deafness-associated loci in three genes is shown in Table 3. In total, 4289 newborns were found to carry at least one pathogenic variant. In the nuclear genome, 23 cases were homozygous for the risk allele, and 4030 were heterozygous. Among the cases carrying MT-RNR1 mutations, 218 were homoplasmic for the risk allele, and 22 were heteroplasmic. The total mutation frequency was 3.01% in this study. GJB2 c.235delC was the most prevalent variant (1.89%), contributing about 62.8% of the total carrier frequency. SLC26A4 c.919-2A>G presented a carrier frequency of 0.98%. The most frequent variant of the MT-RNR1 gene was m.1555A>G, with a carrier frequency of 0.154%, while m.1494C>T was the rarest allele, and had a frequency of 0.015%.

**Table 3:** Spectrum of four deafness associated alleles in 142,417 neonates.

Gene	Cases counts	Carrier frequency* (%)
**GJB2** c.235delC heterozygous	2677	1.890
c.235delC homozygous	16	
**SLC26A4** c.919-2A>G heterozygous	1386	0.978
c.919-2A>G homozygous	7	
**MT-RNR1** m.1555A>G heteroplasmic	22	0.154
m.1555A>G homoplasmic	197	
m.1494C>T heteroplasmic	0	0.015
m.1494C>T homoplasmic	21	
**Total**	4289	3.012

* Carrier frequency is calculated as the frequency of cases either heterozygous or homozygous for the minor allele.

37 neonates carried multiple screened variants. GJB2 c.235delC was the variant most frequently found in combination with other variants (Table 4): 33 neonates were GJB2 c.235delC heterozygous and SLC26A4 c.919-2A>G heterozygous, 3 neonates were GJB2 c.235delC heterozygous and m.1494C>T mutation, and one neonate was SLC26A4 c.919-2A>G heterozygous and m.1555A>G.

**Table 4:** Frequency of compound-mutations among 142,417 neonates.

Mode of compound mutations	Cases counts	Frequency (%)
c.235delC heterozygous vs c.919-2A>G heterozygous	33	0.023
c.235delC heterozygous vs m.1494C>T homoplasmic	3	0.002
c.919-2A>G heterozygous vs m.1555A>G homoplasmic	1	0.0007

### Sequencing validation

A total of 4289 neonates were found to be carriers of at least one of the four variants via real time PCR. The targeted regions of these variants were amplified using appropriate specific primers and sequenced in duplicate via Sanger sequencing. Approximately 53 samples were found to not carry a mutation when sequenced, giving a concordance of 98.7% (1-53/4289).

### Comparison of deafness genetic screening in different cities of China

Studies in more than 20 Chinese cities have assayed common loci associated with NSHL. We compared the results of this study with those of several studies, collected in cities or provinces in China from 2007 to 2015 (Table 5). The total mutation carrier frequency of four variants varied from 1.54% to 5.21%. The frequency identified in our study was 3.01%, slightly lower than the average mutation rate (3.24%). The overall carrier rates reported in 16 areas of China were significantly different via a chi-square test (p<2.2e-16), though large differences were primarily found in cities with relatively small studies.

**Table 5:** Comparison of carrier frequency of mutations associated with hearing loss in 21 cities in China.

Cities	Case counts	Screening period	GJB2 c.235delC			SLC26A4 c.9192A>G			12S rRNA m.1494C>T			12S rRNA m.1555A>G			Total		references
			Homozygote	Heterozygote	Total	Homozygote	Heterozygote	Total	Homoplasmic	Heteroplasmic	Total	Homoplasmic	Heteroplasmic	Total	Total positive case	Frequency of Carrier(%)	
**Wuhan**	142417	May 2014-Dec 2015	16	2677	2693	7	1386	1393	21	0	21	197	22	219	4326	3.01	
**Hefei**	2363	Sept 2014-Dec 2014	0	53	53	0	40	40	0	0	0	5	0	5	98	4.15	[39]
**Kaifeng**	9038	Jan 2013-Mar 2014	3	159	162	3	79	82	1	0	1	3	17	20	265	2.93	[40]
**Luoyang**	2788	Nov 2007-Aug 2008	1	40	41	0	34	34	0	0	0	-	-	6	81	2.91	[41]
**Liaocheng**	11046	Dec 2013-Dec 2014	3	229	232	3	162	165	0	1	1	32	2	34	432	3.91	[42]
**Jinan**	646	Nov 2010-Oct 2011	-	13	13	-	8	8	-	-	2	-	-	2	25	3.87	[43]
**Beijing**	89924	Oct 2012-Sept 2014	9	1693	1702	7	1197	1204	11	1	12	135	45	180	3098	3.45	[44]
**Changzhi**	19113	Jun 2013-Dec 2013	2	341	343	0	370	370	-	-	4	-	-	65	782	4.04	[45]
**Tianjin**	58397	Dec 2011-Dec 2012	8	1135	1143	7	902	909	8	0	8	84	17	101	2161	3.7	[30]
**Shijiazhuang**	10948	Jan 2014-Aug 2015	1	182	183	1	155	156	0	0	0	17	3	20	358	3.27	[46]
**Foshan**	10238	May 2012-Dec 2013	4	165	169	3	82	85	1	0	1	18	4	22	277	2.71	[47]
**Shaoguan**	863	2014	0	12	12	0	24	24	0	2	2	5	7	12	48	5.56	[48]
**Suzhou**	5800	Oct 2011-Feb 2012	-	-	109	-	-	94	-	-	1	-	-	8	212	3.66	[49]
**Wuxi**	2553	Jan 2013-Dec 2013	0	67	67	1	21	22	0	0	0	3	1	4	93	3.64	[50]
**Nantong**	765	Jan 2014-Apr 2014	-	-	25	-	-	12	1	-	1	0	0	0	38	4.97	[51]
**Yangzhou**	965	Apr 2013-Aug 2014	2	30	32	0	16	16	0	0	0	-	-	1	49	5.08	[52]
**Shaoxing**	5121	Jun 2013-Dec 2013	2	111	113	0	60	60	0	0	0	6	0	6	179	3.5	[53, 54]
**Nanning**	10224	Jan 2014-Jun 2015	3	131	134	2	46	48	0	0	0	20	4	24	206	2.01	[55, 56]
**Chengdu**	17000	Aug 2012-Jun 2013	2	254	256	0	130	130	4	0	0	34	8	42	431	2.54	[57]
**Gansu**	10043	Dec 2009-Apr 2010	2	117	119	0	93	93	-	-	3	-	-	16	230	2.29	[58]
**Xinjiang**	1038	-	0	4	4	-	9	9	-	-	-	3	0	3	16	1.54	[59]
**Total**	411290	-	59	7362	7555	35	4776	4917	49	4	59	565	127	790	13320	3.24	

### Method comparison of qPCR with ARMS-PCR

We used real-time PCR with Taqman technology and ARMS-PCR to assay the variant sites simultaneously in 707 neonates. The results for GJB2 c.235delC, m.1555A>G, m.1494C>T in these 707 newborns showed complete accordance. With SLC26A4 c.919-2A>G, 378 samples were consistent between methods, the remaining 314 newborns had no results at this site with the ARMS-PCR (Table 6). Three rounds of PCR were attempted if assays failed to produce detectable product (except for SLC26A4 c.919-2A>G, which demonstrated such a high failure rate with ARMS-PCR that efforts to evaluate it with this method were discontinued after the first round). Real-time PCR had a substantially lower failure rate than ARMS-PCR (Tables 7 and 8).

**Table 6:** Genotype results comparison of the PCR methods.

			**ARMS-PCR**										
			GJB2			SLC26A4			MT-RNR1				Total
			c.235delC Heterzygous	c.235delC Homozyous	Wild type	c.919-2A>G Heterzygous	c.919-2A>G Homozyous	Wild type	m.1555 A>G Wild type	m.1555 A>G Mutation type	m.1494 C>T Wild type	m.1494 C>T Mutation type	
**Taqman PCR**	GJB2	c.235delC Heterzygous	8	0	0								707
		c.235delC Homozyous	0	0	0								
		Wild type	0	0	699								
	SLC26A4	c.919-2A>G Heterzygous				4	0	0					378
		c.919-2A>G Homozyous				0	0	0					
		Wild type				0	0	374					
	MT-RNR1	m.1555 A>G Wild type							705	0			707
		m.1555A>G Mutation type							0	2			
		m.1494 C>T Wild type									707	0	707
		m.1494 C>T Mutation type									0	0	
	Total		707			378			707		707		

**Table 7:** Failure rate of ARMS-PCR method.

	SLC26A4	GJB2	m.1555 A>G	m.1494 C>T
**Number assayed**	692	707	707	707
**Not- detected (first round)**	314	38	16	18
**Not-detected (second round)**	NA	16	3	8
**Not-detected (third round)**	NA	1	2	0

**Table 8:** Failure rate of real-time PCR.

	SLC26A4	GJB2	m.1555 A>G	m.1494 C>T
Number assayed	707	707	707	707
Not- detected (first round)	5	3	1	0
Not-detected (second round)	0	0	0	0

The time required for real time PCR was significantly lower than for ARMS-PCR, primarily due to differences in the requirements for DNA preparation and visualization of the amplification product. Processing each batch of ARMS-PCR entailed in total six hours, vs two hours for real time PCR. In addition, the reagents costs were 25% higher for ARMS-PCR.

## Discussion

Hearing loss is a relatively common disorder, featured by high heterogeneity and phenotypic diversity. Early identification of infants with NSHL can avoid social and language difficulties that can occur in infants with undiagnosed hearing loss[8, 60, 61]. Universal programs to screen newborns for hearing defects are greatly aiding the identification of children with hearing loss, however estimates of the test failure rates range from 2 to 4 percent, and the tests can have poor specificity[4]. Additionally, late onset hearing loss and deafness induced by ototoxicity cannot be identified by these programs. One study of physical screening of newborns found that almost all carriers of 12S rRNA mutations passed the physical test[62]. Besides aiding in improved sensitivity for the detection of newborns with severe hearing loss, genetic testing could support the use of physical testing for newborns with only moderate hearing loss by detecting newborns with greater risk of hearing loss, and thus help avoid the trade-off of improved sensitivity for decreased specificity. Genetic screening, as a compliment to physical hearing assessments, could be an important aid in managing this disorder in children.

More than 70 genes have been identified to harbor variations linked to non-syndromic hearing loss. Previous studies have identified GJB2 as in important contributor to hereditary NSHL, and mutations in GJB2 can be detected in nearly 50% of patients with autosomal recessive hearing loss[63]. The c.235delC mutation rate was 18.16% in 1680 cases of Chinese NSHL patients from 13 provinces. Hearing loss related to single-site mutations is relatively infrequent and is unlikely to account for the heterogeneity of a disorder like hearing loss[64]. In this study, 2693 neonates were found to carry 235delC mutations (1.9% of all cases), and we found that c.235delC alleles are the most frequent compound mutations, accounting for 0.025% (36 cases) of all the neonates. About 80% of large vestibular aqueduct syndrome patients have been found to harbor SLC26A4 c.919-2A>G mutations[65]. In this study we found that 0.98% of neonates were c.919-2A>G carriers. SLC26A4 c.919-2A>G compound heterozygosity in hearing-impaired patients is common[66], this study found one case to carry that variant and the mitochondrial A1555G variant.

The m.1555A>G and m.1494C>T are the main pathogenic mutations of 12S rRNA gene. These rare variants have been shown to have a direct relationship with aminoglycoside induced hearing loss[67]. There were no heteroplasmic cases and 21 neonates were homoplasmic for m.1494C>T variant, while 10.0% of m.1555A>G variants occurred as heteroplasmic mutations (22/219). Mitochondrial heteroplasmy can occur at various rates in different populations[68]. Heteroplasmy of m.1555A>G has been observed as an uncommon phenomenon in both European and Asian populations [69–72]. Other studies have found that individuals with a higher proportion of m.1555A>G variants were more likely to exhibit hearing loss[69, 70].

Several methods for the detection of genetic variants related to hearing loss have been employed in the clinic. ARMS-PCR offers consistent and accurate results with inexpensive equipment. However, the running time for every target is about three hours. Additionally, different loci require different reaction conditions with this methodology. MALDI-TOF mass spectrometry high-throughput genotyping [73] and chip based assays can be effective[74], but can entail high costs. Therefore, additional technologies that allow efficient, flexible, and high throughput genotyping with low cost barriers to entry would likely facilitate the adoption of genetic screening, particularly in developing regions. We found that real-time PCR based on Taqman technology, which can be performed in a conventional real-time PCR machine, can be an effective method for genetic screening in a community health care environment. This method, using fluorescently labeled allele-specific probes, allows rapid and reliable detection of DNA mutations, including single nucleotide polymorphisms, insertions, and deletions. 384 samples can be assayed on one machine in only 1.5 hours, and two hours are required to prepare samples, thus the procedure is suitable for large-scale screenings. Additionally, we found that lower amounts of reagents are required for real-time PCR compared to conventional PCR. Finally, the failure rate of the real time PCR was lower than that of conventional PCR as well. All real-time PCR assays which failed in the first attempt succeeded in the second attempt, while ARMS-PCR failed several times after even three attempts.

The next stage of this study is a comparison between the variants identified via PCR and the results of physical hearing screening. The neonates carrying the positive variants will be tested again by otoacoustic emission testing or automated auditory brainstem response to identify associations with late onset hearing loss. In addition, it is hoped that the identification of alleles which are contraindicative for the use of aminoglycoside antibiotics will prevent their use. Further collaboration with the participating hospitals will determine the specificity and sensitivity of the association of the studied variants with hearing loss at birth and arising in early childhood, allowing an estimation of costs and benefits of delivering newborn hearing genetic screening in a large-scale community setting.

## Supporting information

S1 Table Anonymized genotype results are included as supplementary file 1.xlsx. (XLSX)
